# Effect of Sintering Temperature on the Properties of Highly Electrical Resistive SiC Ceramics as a Function of Y_2_O_3_-Er_2_O_3_ Additions

**DOI:** 10.3390/ma13214768

**Published:** 2020-10-26

**Authors:** Sheng Ge, Xiumin Yao, Yingying Liu, Hang Duan, Zhengren Huang, Xuejian Liu

**Affiliations:** 1 Shanghai Institute of Ceramics, Chinese Academy of Sciences, Shanghai 200050, China; gesheng@student.sic.ac.cn (S.G.); liuyingying@student.sic.ac.cn (Y.L.); duanhang@student.sic.ac.cn (H.D.); 2 University of Chinese Academy of Sciences, Beijing 100049, China

**Keywords:** SiC ceramics, spark plasma sintering, electrical resistivity, thermal conductivity

## Abstract

Silicon carbide (SiC) ceramics with Y_2_O_3_-Er_2_O_3_ as sintering additives were prepared by spark plasma sintering (SPS). The effects of sintering temperatures and Y_2_O_3_-Er_2_O_3_ contents on the microstructure, thermal conductivity, electrical, and mechanical properties were investigated. The increasing of sintering temperatures promoted the densification of SiC ceramics, thus increasing the thermal conductivity and electrical resistivity. With the increase of the sintering additive contents, the electrical resistivity increased due to the formation of the electrical insulating network; and the thermal conductivity first increased and then decreased, which was related to the content and distribution of the secondary phase among the SiC grains. The SiC ceramics sintered at 2000 °C with 9 wt.% Y_2_O_3_-Er_2_O_3_ exhibited higher electrical resistivity and thermal conductivity, which were 4.28 × 10^9^ Ω·cm and 96.68 W/m·K, respectively.

## 1. Introduction

Silicon carbide (SiC) ceramic has enjoyed a good reputation for its excellent mechanical, chemical, and thermal properties [1,2,3,4]. It has been widely used as structural materials, such as mechanical seals [5], heat exchangers [6], and optical mirrors for space telescopes [7]. However, it has more applications besides structural materials. Nowadays electronic packaging technology is developing towards higher voltage, larger current, and greater power, which presents great challenges to the ceramic substrates [8]. At present, AlN and Si_3_N_4_ are commonly used as substrate materials, but their wide applications are limited due to their disadvantages. For example, AlN has low high-temperature strength and fracture toughness [9], and Si_3_N_4_ shows insufficient thermal conductivity [10]. If the electrical resistivity of SiC materials can be increased while maintaining the high thermal conductivity, SiC would undoubtedly be a suitable substrate material. Therefore, how to increase the electrical resistivity of SiC ceramics is particularly important.

Lots of efforts have been made to increase the electrical resistivity of SiC ceramics. Kim et al. [11] reported hot-pressing sintering SiC ceramics doped with 3 vol.% AlN-Y_3_Al_5_O_12_(YAG) exhibited high electrical resistivity, as high as 1.3 × 10^10^ Ω·cm. They pointed out that such high resistivity was ascribed to Al_2_O_3_, in which Al impurities substituting Si site acted as deep acceptors for trapping carriers. SiC-BN composites with high resistivity, 4.11 × 10^11^ Ω·cm, were achieved by pressureless solid-state sintering companying with in situ synthesis process [12]. Interface diffusion of B and N from BN into SiC was crucial to improving the insulating and dielectric properties through carrier compensation. Liang et al. [13] obtained SiC ceramics with a high electrical resistivity of 3.52 × 10^11^ Ω·cm by SPS with Al_2_O_3_ and Er_2_O_3_ as sintering additives. They attributed the high electrical resistivity to the interconnection and the amorphous nature of the grain boundary phase. The above works have obtained high-resistance SiC ceramics, but the thermal conductivity is insufficient, which cannot meet the requirements of substrate materials.

It is well-known that a clean crystal lattice is beneficial for improving the thermal conductivity of the material [14,15]. Compared to common metal oxides sintering additives, such as Al_2_O_3_, the rare earth oxides are difficult to enter the SiC crystal lattice due to their large ion radius. Besides, rare earth oxides can also react with SiO_2_ on the surface of SiC particles, to form the liquid phase, which is conducive to the process of densification. The existence of the liquid phase can also greatly increase the grain boundary resistance. In a word, the introduction of rare earth oxides is expected to achieve the high resistivity and high thermal conductivity of SiC ceramics.

In the present work, SiC ceramics doped with Y_2_O_3_-Er_2_O_3_ were prepared by SPS. One advantage of this sintering method is that the grain growth of the SiC ceramic is restricted because of considerably short dwelling time. It has been reported that more grain boundaries are beneficial for improving electrical resistivity [11,16]. The effects of sintering temperature and oxides’ contents on electrical resistivity, thermal conductivity, and mechanical properties of SiC ceramics were explored, and the mechanisms of the enhanced electrical resistivity and increased thermal conductivity were analyzed.

## 2. Experiment Procedure

Commercially available α-SiC (0.64 μm, 98.57% pure), Er_2_O_3_, and Y_2_O_3_ were used as the starting powders in the present work. The relative content of sintering additive powders in these batches was 1, 3, 5, 7, and 9 wt.%. The molar ratio between Er_2_O_3_ and Y_2_O_3_ was 1:1. At the first, five batches of powders were milled in ethanol for 4 h, using SiC grinding balls at the speed of 300 rpm (shown in Table 1). After milling, the powders were dried in a drying oven at 60 °C for 12 h. Then the mixture was sieved through a 100-screen sieve. In the end, the obtained powders were put into a graphite die (20 mm in diameter) and sintered with the SPS system (SPS, PS, Dr. Sinter 2020, Sumi-tomo Coal Mining Co., Tokyo, Japan), in a vacuum. The heating and cooling rates were both 100 K·min^−1^, and the pressure was 30 MPa. The samples were all held for 10 min at sintering temperatures (1800, 1850, 1900, 1950, and 2000 °C).

The bulk densities of the samples were measured by the Archimedes method. The theoretical densities were calculated according to the rule of mixtures, as shown in Table 1. The phase compositions were analyzed by standard powder X-ray diffraction (XRD, D/Max-2250V, Rigaku, Tokyo, Japan). Scanning electron microscope (SEM, Magellan 400, FEI, Hillsboro, American) was used to observe the microstructures of the polished surfaces. The Vickers hardness was determined with a load of 9.8 N and a dwell time of 10 s. The fracture toughness was estimated by the crack lengths and indentation diagonal lengths.

For thermal conductivity measurement, SiC ceramics were processed into a disk-like shape (10 mm in diameter and 2.5 mm thick). The thermal diffusivity and specific heat measurements were measured by a laser-flash apparatus (LFA447 Nanoflash, NETZSCH Instruments Co. Ltd., Selb, Germany). Then thermal conductivity (κ) was calculated from the equation κ = ραCp. For electrical resistivity measurement, samples were processed into a disk-like shape (10 mm in diameter and 2 mm thick). Then the silver electrodes were pasted on both sides of the samples. The direct current (dc) electrical resistivity was measured on a high-resistance meter (Model HP4329A, Hewlett-Packard, Palo Alto, CA, USA) with an applied voltage of 10 V.

For further microstructure observation, SiC ceramic sample was cut into 3 mm diameter and 100 μm in thickness, for a transmission electron microscope (TEM, Technai G2 F20, FEI, USA) test. Then it was ion-beam-thinned to about 10 μm in thickness. Moreover, an energy-dispersive spectrometer (EDS) was used to analyze the composition of the sample.

## 3. Results and Discussion

### 3.1. Phase Composition

**Figure 1:**
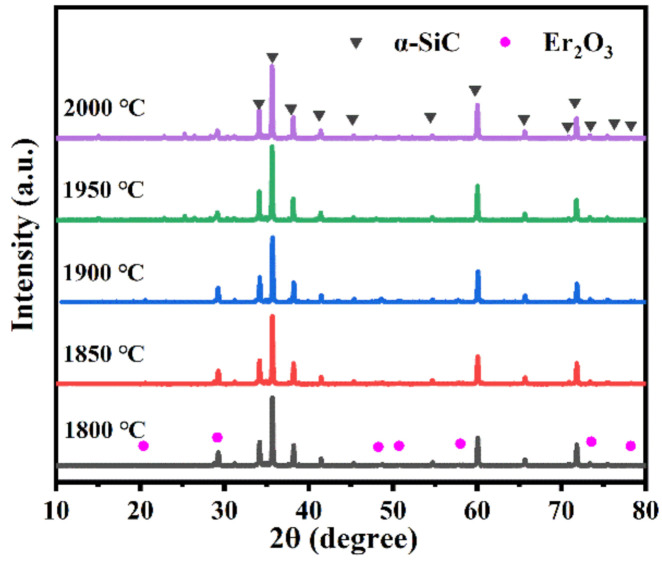
XRD patterns of SiC ceramics sintered at different temperatures.

Figure 1 shows the XRD patterns of the SiC ceramics with different sintering temperatures for adding 9 wt.% Y_2_O_3_-Er_2_O_3_. The α-SiC was the main phase, while it contained a small amount of Er_2_O_3_. However, the Y_2_O_3_ phase was not found in the XRD patterns. It may be that the Y_2_O_3_ is easier to form an amorphous phase with SiO_2_ of the SiC surface [17] than Er_2_O_3_ during the sintering process. From the XRD patterns of SiC ceramics sintered at different temperatures, it can be inferred that there were no other crystallized phases except for the SiC and additives. The rapid cooling rate resulted in the formation of the amorphous phase, which would cause phonon to strongly scatter [15].

**Figure 2:**
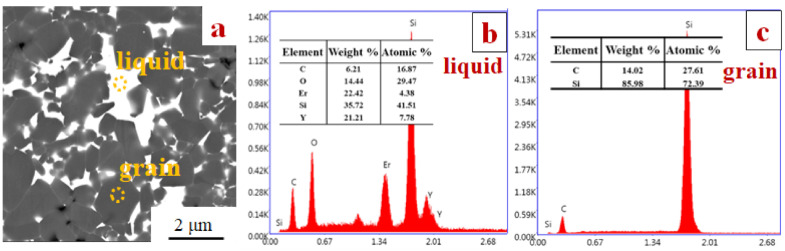
The microstructure and EDS results of SiC ceramics: (**a**) SEM, (**b**) EDS result for the secondary phase, and (**c**) EDS result for the grain.

Figure 2 shows the typical microstructure and EDS results of SiC ceramics. From the picture, the gray continuous phase was the liquid phase, whereas the dark well-dispersed particulate phase was SiC. It can be seen that the particle sizes of SiC grains were below 2 µm. The intergranular phases were formed among SiC grains. EDS results (Figure 2b) show that the compositions of intergranular phases were O, Si, Y, Er, and C elements. However, the XRD analysis reveals that α-SiC and Er_2_O_3_ were the main crystalline phases, and no crystalline phases containing O, Si, Y, Er, and C elements were detected, which implies that the intergranular phases were composed of an amorphous C-O-Si-Y-Er phase. There are three possible processes for this result. (1) The Y_2_O_3_-Er_2_O_3_ additives reacted with SiO_2_ film on the surface of SiC particles [17], to form an amorphous O-Si-Y-Er melt. (2) An amorphous C-O-Si-Y-Er melt was formed from the dissolution of SiC particles into the O-Si-Y-Er melt. (3) SiC samples were densified by the C-O-Si-Y-Er melt via liquid-phase sintering and remained at the end of the sintering because of the rapid cooling at a rate of 100 K·min^−1^.

**Figure 3:**
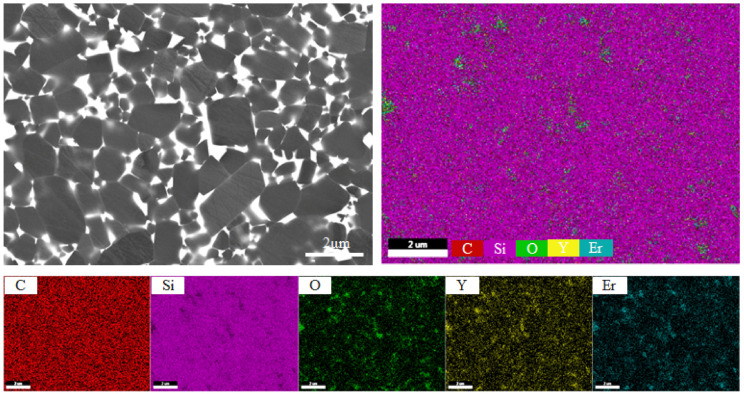
Typical EDS mapping of SiC samples.

For exploring the distribution uniformity of these elements, the EDS mapping of the typical sample was observed in Figure 3. The distribution of Si and C elements was significantly uniform, whereas the distribution of Y, Er, and O elements in the samples was relatively uneven; moreover, the O element matched quite well with the Y element and Er element in this area, indicating that the C-O-Si-Y-Er phase was formed in the sample. Apart from this, the uneven distribution of the Y, Er, and O elements indicates that the SiC particles were not surrounded uniformly by the second phase, which might be harmful to the electrical property of the samples.

### 3.2. The Influence of Sintering Temperature

#### 3.2.1. Microstructure

**Figure 4:**
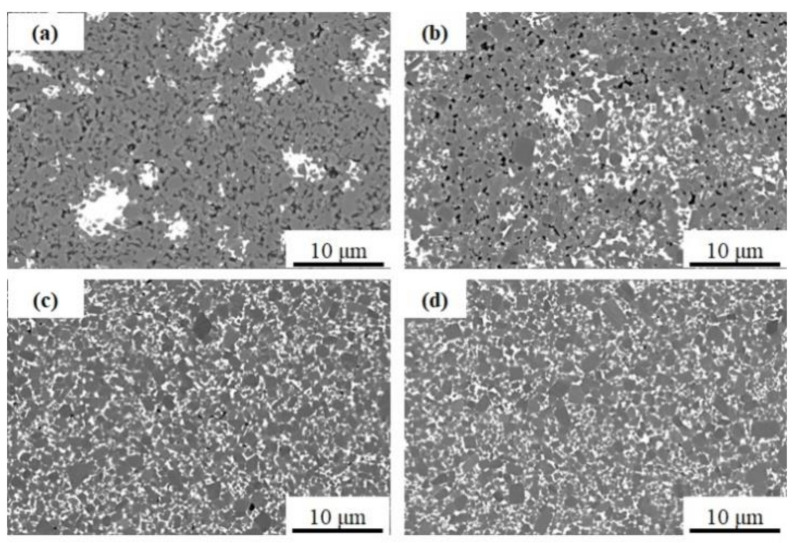
Polished surfaces of sintered SiC samples with 9 wt.% Y_2_O_3_-Er_2_O_3_ at different sintering temperatures: (**a**) 1850 °C, (**b**) 1900 °C, (**c**) 1950 °C, and (**d**) 2000 °C.

The microstructures of the SiC samples sintered at different temperatures are shown in Figure 4. As the sintering temperature increased, more liquid phases were formed, and the distribution of them was more uniform. Apart from this, the pores in the samples decreased, and the SiC particles gradually grew up. At 2000 °C, there were few pores, and the average grain size of SiC particles was about 2 μm.

#### 3.2.2. The Thermal Conductivity

**Figure 5:**
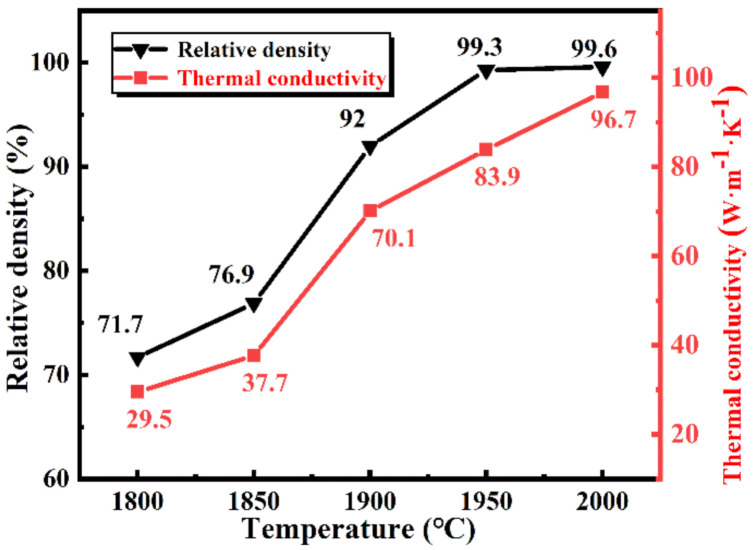
Relative density and thermal conductivity of the sintered SiC samples with 9 wt.% Y_2_O_3_-Er_2_O_3_ as a function of sintering temperatures.

As detailed in Figure 5, as the sintering temperature increases from 1800 to 2000 °C, the relative density of the samples increases significantly from 71.72% to 99.6%, which is in agreement with the results in Figure 4. Apart from this, the thermal conductivity of the samples varies significantly from 29.46 to 96.68 W·m^−1^·K^−1^, which shows a similar trend with the relative density. The thermal conductivities of SiC ceramics sintered at 1800 and 1850 °C are extremely lower. There are two possible reasons for this: (1) the existence of a large number of pores, or (2) the small grain size of SiC particles. It is known that the existence of porosity and grain boundaries in ceramics greatly decreased thermal conductivity [14]. When the sintering temperature was over 1900 °C, the grain size of SiC particles increased, and pores decreased sharply. Apart from this, the distribution of the secondary phase was more uniform. All of them show a positive effect on the thermal conductivity of the samples and lead to an increase in thermal conductivity. Compared to the samples sintered at 1950 and 2000 °C, the grain size of SiC particles was almost the same, and the relative density of the samples was only increased by 0.34%. However, the thermal conductivity increased by 15.2%, which indicated that the pores in the samples were obviously influenced by the samples’ thermal conductivity.

#### 3.2.3. The dc Resistivity

**Figure 6:**
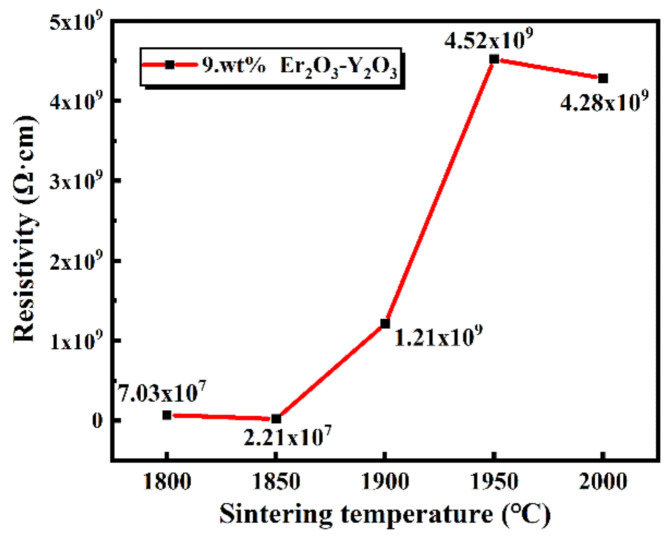
The dc electrical resistivity of the sintered SiC samples with 9 wt.% Y_2_O_3_-Er_2_O_3_ as a function of the sintering temperatures.

As evident in Figure 6, with the increase of sintering temperature, the dc resistivity of the samples rapidly increases before 1950 °C, whereas it decreases slightly after 1950 °C. The possible reason was that the liquid phases were gradually formed but not evenly distributed among the SiC grains when the temperature was below 1900 °C. Besides, there were many pores in the samples. Due to the uneven distribution of the liquid phase, SiC grains connected, and the samples exhibited semiconductor characteristics. When the temperature increased, a more liquid phase was formed and evenly distributed among the SiC grains, which prevented the contact of SiC particles (seen in Figure 4c,d). Finally, the resistivity of SiC ceramics sintered after 1900 °C increased. Moreover, the highest electrical resistivity, about 4.52 × 10^9^ Ω·cm, was achieved for the SiC ceramic sintered at 1950 °C. With the further increase of the temperature, part of the liquid phases will evaporate. In this case, some SiC grains cannot be surrounded by the liquid phase well, which results in a slight decrease in electrical resistivity of the samples sintered at 2000 °C.

#### 3.2.4. The Hardness and Fracture Toughness

**Figure 7:**
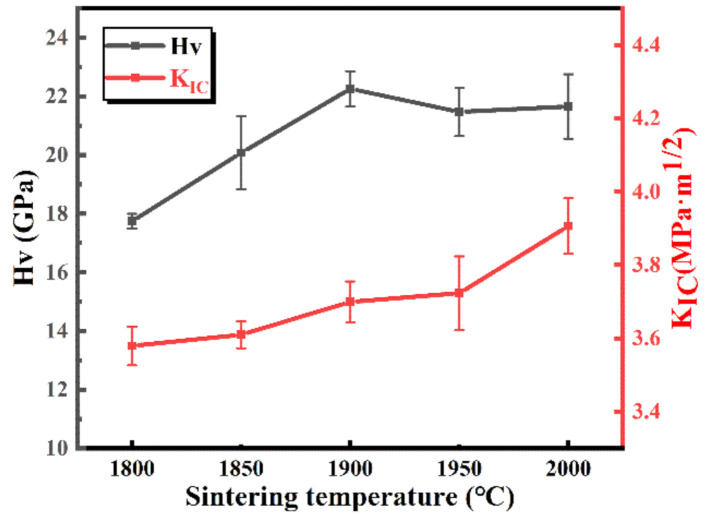
The hardness and fracture toughness of the sintered SiC samples with 9 wt.% Y_2_O_3_-Er_2_O_3_ as a function of the sintering temperatures.

The Vickers hardness (H_V_) and fracture toughness (K_IC_) of SiC ceramics as a function of the sintering temperature are displayed in Figure 7. The fracture toughness of the samples gradually increased as the temperature increased, which was related to the secondary phase. From Figure 4, it can be found that, with the increase of temperature, the secondary phase of the ceramics was distributed more evenly, which was beneficial for the fracture toughness. The hardness of the samples firstly increased as the sintering temperature increased to 1900 °C; the reason for the increase was due to the increase of the density (in Figure 5). Then, although the densities of the samples were increased to 99%, the hardness of the ceramics reduced a little and retained at about 4.2 GPa; it may be the formation of much liquid phase.

### 3.3. The Influence of Sintering Additive Content

#### 3.3.1. Microstructure

**Figure 8:**
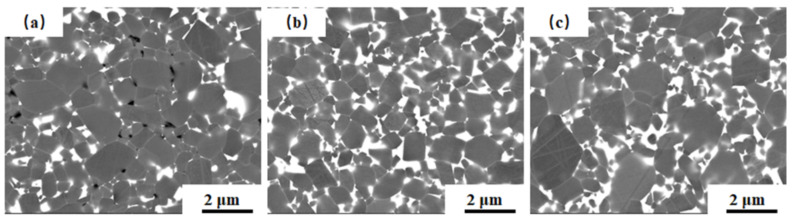
Polished surfaces of sintered SiC samples at 1950 °C, with different sintering additives contents: (**a**) 5 wt.%, (**b**) 7 wt.%, and (**c**) 9 wt.%.

Polished surfaces of sintered SiC samples with different sintering additives contents are shown in Figure 8. It can be seen that, with the increase of sintering additives contents, the liquid phase among SiC grains increases, and the pores decrease. There were almost no pores in the samples sintered with 7 wt.% sintering additives, and the distribution of the liquid phase more even. High densification is beneficial for improving the thermal conductivity; even liquid phase distribution is beneficial for improving the electrical resistivity.

#### 3.3.2. The Thermal Conductivity

**Figure 9:**
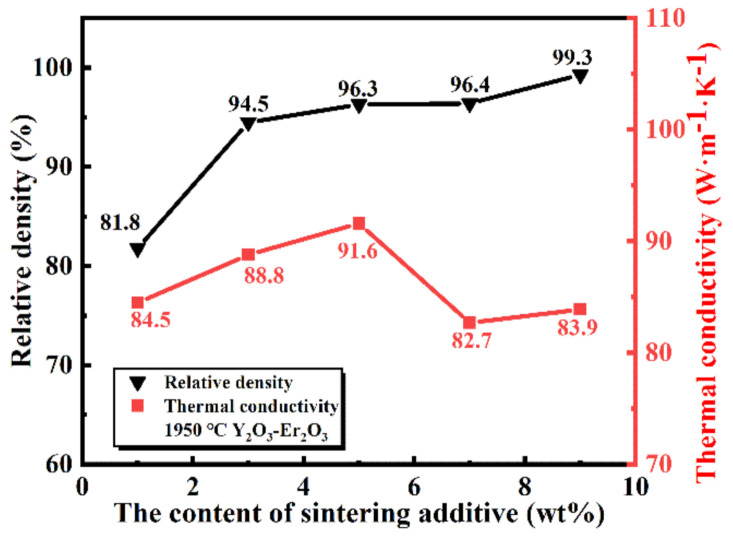
Relative density and thermal conductivity of the sintered SiC samples at 1950 °C as a function of the sintering additives contents.

Relative density and thermal conductivity of the sintered SiC samples as a function of the sintering additive contents are shown in Figure 9. It is found that the relative density of the samples increased as the sintering additives contents increased, which is in agreement with the results in Figure 8. According to the calculation, the samples were almost densified with the relative density higher than 96.2%.

However, the thermal conductivity of the samples firstly increased and then decreased. The maximum value was obtained in the samples containing 5 wt.% sintering additives. Firstly, the reason for the increase in thermal conductivity can be attributed to the increase in relative density. Secondly, with the further increase of sintering additive contents, the content of the liquid phase increased, which prevents the contact of SiC particles by liquid phase (seen in Figure 8). Apart from this, the liquid phase has lower thermal conductivity than that of the SiC phase, and both of them led to a decrease in the thermal conductivity of the samples.

#### 3.3.3. The dc Resistivity

**Figure 10:**
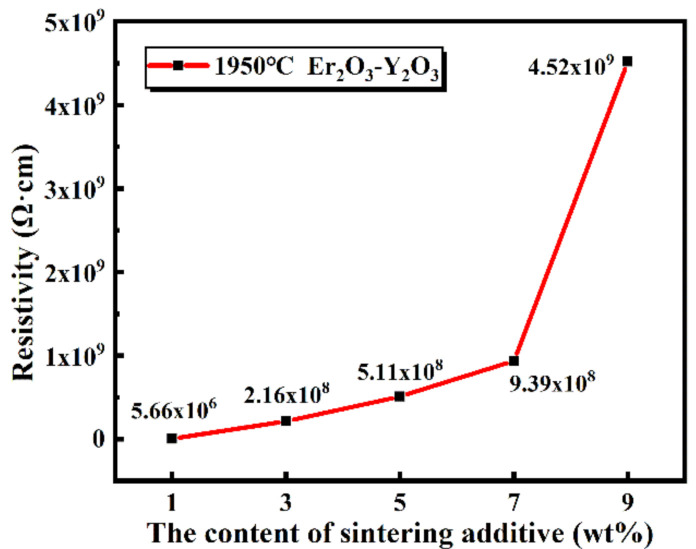
The dc electrical resistivity of the sintered SiC samples at 1950 °C as a function of the sintering additive contents.

The data in Figure 10 suggest that the sintering additives’ content has an important influence on the electrical resistivity of the samples. The electrical resistivity of the SiC ceramics increased significantly with the increase of the sintering additive contents. It is worth noting that, when the sintering additive content just changed from 7 to 9 wt.%, resistivity varied markedly from 9.39 × 10^8^ Ω·cm to 4.52 × 10^9^ Ω·cm. The content of the liquid phases in the samples increased with the increase of the sintering additive contents, and liquid phases were also distributed more evenly (shown in Figure 8). The liquid phases were effective barriers for discouraging carrier transport between the SiC grains. Therefore, the SiC grains were surrounded by the insulating liquid phase, and the electrical insulating network was formed. This is the possible reason for the increase of the electrical resistivity.

#### 3.3.4. The Hardness and Fracture Toughness

**Figure 11:**
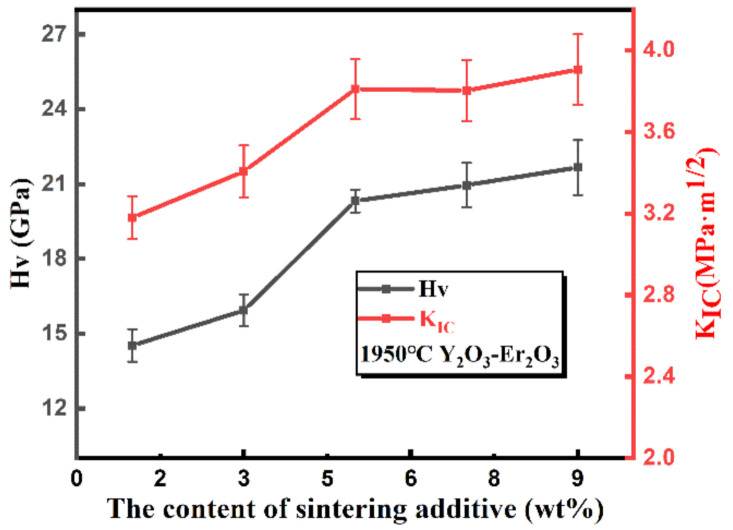
The hardness and fracture toughness of the sintered SiC samples at 1950 °C as a function of the sintering additive contents.

The hardness and fracture toughness of the sintered SiC samples as a function of the sintering additive content are shown in Figure 11. It indicates that the hardness and fracture toughness of samples both increased when the content of Y_2_O_3_-Er_2_O_3_ increased, possibly because of the increase of the relative density. The increase of the secondary-phase content promoted the densification of ceramic samples. The hardness and the fracture toughness of the samples with 9 wt.% Y_2_O_3_-Er_2_O_3_ approached to above 21 GPa and 3.8 MPa·m^1/2^, respectively.

### 3.4. The Influence of the Microstructure

In order to analyze the influence of the microstructure on the electrical resistivity and thermal conductivity of SiC ceramics samples, TEM was used to observe the morphologies of SiC–SiC grain boundaries and the triple junction boundaries. The SiC sample sintered at 2000 °C with 9 wt.% Y_2_O_3_-Er_2_O_3_ was chosen for observation because it had good overall properties.

**Figure 12:**
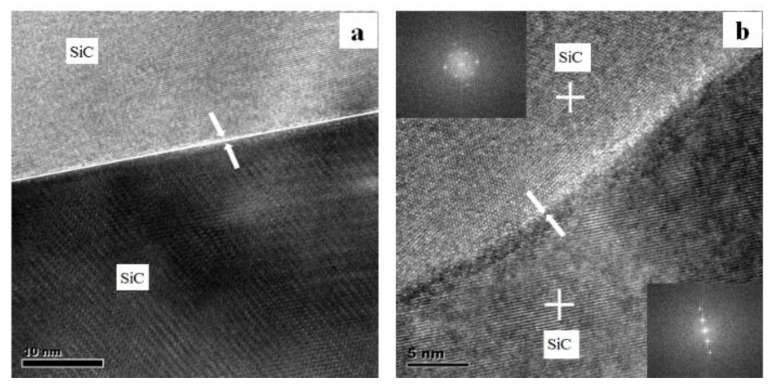
HRTEM image shows the SiC–SiC grain boundary of the SiC ceramic sample with EY9 sintered at 2000 °C with (**a**) amorphous film and (**b**) clean grain boundary.

After checking most of the SiC–SiC grain boundaries carefully, the typical ones are shown in Figure 12. It can be observed that there were two types of grain boundaries in the sample, including amorphous films and clean grain boundaries. Most of them were amorphous films (shown in Figure 12a), which implied the interconnected network formation of the second phase in the sample. It is known from the literature that the resistivity will increase when the conduction path between the SiC grains is disconnected by the insulating glass phase [18]. This may be the reason for the dc resistivity of the SiC sample as high as 4.28 × 10^9^ Ω·cm. However, clean boundaries were not uncommon. It is believed that clean grain boundaries are beneficial to increase the thermal conductivity of the SiC ceramics [19]. Moreover, the clean grains’ boundaries would allow the carrier to transport between the SiC grains, which is extremely harmful to increase the resistivity.

**Figure 13:**
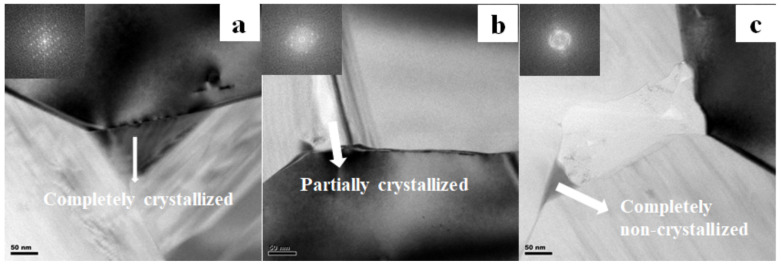
Different triple junction grain boundaries of the SiC ceramic sample with EY9 sintered at 2000 °C: (**a**) completely crystallized, (**b**) partially crystallized, and (**c**) completely non- crystallized.

**Figure 14:**
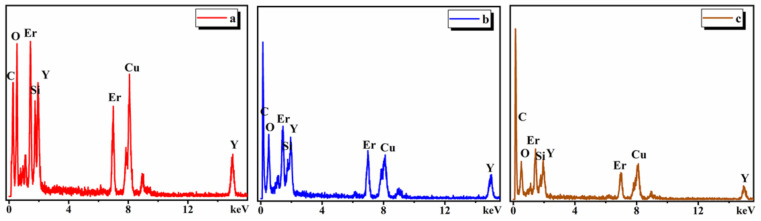
Different EDS analysis of the triple junction grain boundary: (**a**) completely crystallized, (**b**) partially crystallized, and (**c**) completely non- crystallized.

Three types of triple junction grain boundaries were found in the SiC sample (shown in Figure 13), i.e., completely crystallized, partially crystallized, and completely non-crystallized triple junction boundaries. Moreover, EDS analysis of different types of triple junction boundaries in Figure 14 reveals compositions of the triple junction boundaries phase. It has been reported that the SiC ceramics with high electrical resistivity (3.52 × 10^11^ Ω·cm) sintered by Liang [13] only had a completely non-crystallized triple junction boundary phase. Therefore, the existence of three kinds of triple junction grain boundaries in SiC samples should also be responsible for the relatively low electrical resistivity.

## 4. Conclusions

SiC ceramics with different Y_2_O_3_-Er_2_O_3_ additive contents (1~9 wt.%) were prepared by SPS at 1800~2000 °C. The effects of additive contents on the microstructure and properties of SiC ceramics were studied. The XRD results show that the main phases of the samples are SiC and Er_2_O_3_. The thermal conductivity and electrical resistivity of the samples increase with the increase of sintering temperature, mainly due to the reduction of porosity and more evenly distribution of the secondary phase. With the increase of the sintering additive content, the content and distribution of the secondary phase among the SiC grains influence the formation of the conductive network. While the content of the additive is 9 wt.%, SiC ceramics sintered at the temperature of 2000 °C exhibited high electrical resistivity (4.28 × 10^9^ Ω·cm), good thermal conductivity (96.68 W/m·K), great hardness (21.7 GPa), and modest fracture toughness (3.9 MP·m^1/2^) at room temperature.

## Figures and Tables

**Table 1:** Batch composition and theoretical density of SiC ceramics samples.

Sample	Batch Composition (wt.%)	Theoretical Density (g/cm^3^)
EY1	99 wt.% SiC + 0.63 wt.% Er_2_O_3_ + 0.37 wt.% Y_2_O_3_	3.212
EY3	97 wt.% SiC + 1.88 wt.% Er_2_O_3_ + 1.12 wt.% Y_2_O_3_	3.252
EY5	95 wt.% SiC + 3.14 wt.% Er_2_O_3_ + 1.86 wt.% Y_2_O_3_	3.287
EY7	93 wt.% SiC + 4.40 wt.% Er_2_O_3_ + 2.60 wt.% Y_2_O_3_	3.323
EY9	91 wt.% SiC + 5.67 wt.% Er_2_O_3_ + 3.33 wt.% Y_2_O_3_	3.360
